# Persistent psychogenic déjà vu: a case report

**DOI:** 10.1186/1752-1947-8-414

**Published:** 2014-12-08

**Authors:** Christine E Wells, Chris JA Moulin, Paige Ethridge, Nathan A Illman, Emma Davies, Adam Zeman

**Affiliations:** 7 Department of Psychology, Sociology and Politics, Sheffield Hallam University, Sheffield, SD10 2BQ UK; 8 Université de Bourgogne, LEAD - CNRS UMR 5022, Pôle AAFE, 11 Esplanade Erasme, 21000 DIJON, France; 9 University of Western Ontario, 1151 Richmond St, London, ON N6A 3K7 Canada; 10 Institute of Psychiatry, Kings College London, London, WC2R 2LS UK; 11 Institute of Psychological Sciences, University of Leeds, Leeds, LS2 9JT UK; 12 University of Exeter Medical School, St Luke’s Campus, Exeter, EX11 1LU UK

**Keywords:** Anxiety, Déjà vu, Recognition memory

## Abstract

**Introduction:**

Déjà vu is typically a transient mental state in which a novel experience feels highly familiar. Although extensively studied in relation to temporal lobe epilepsy as part of simple partial seizures, déjà vu has been less studied in other clinical populations. A recent review of temporal lobe epilepsy suggested a possible link between clinical levels of anxiety and debilitating déjà vu, indicating further research is required. Here, for the first time in the literature, we present a case study of a young man with anxiety and depersonalisation who reported experiencing persistent and debilitating déjà vu. This report therefore adds to the limited literature on the relationship between anxiety and déjà vu.

**Case presentation:**

A 23-year-old White British man presented with a form of persistent déjà vu in 2010, approximately 3 years since symptom onset. He reported a history of anxiety and experiencing feelings of depersonalisation. Neurological assessment (electroencephalogram and magnetic resonance imaging) did not indicate any abnormalities. We assessed his recognition memory with a task used in patients with dementia who report similar experiences but lack awareness of their falseness.

**Conclusions:**

Our case’s memory performance was more conservative than controls but did not indicate a memory deficit. Unlike other patients with chronic déjà vu (for example, in dementia), he is fully aware of the false nature of his déjà vu and this presumably leads to his intact recognition memory performance. We suggest that his persistent déjà vu is psychogenic and conclude that déjà vu should be further studied in psychiatric disorders.

## Introduction

Déjà vu is typically a transitory mental state in which a novel experience feels highly familiar. Much of the scientific literature has studied déjà vu in relation to temporal lobe epilepsy (TLE) as part of simple partial seizures [1]. Recent research has shown that déjà vu experiences are of comparable phenomenology and intensity in TLE [2]. In both participants with epilepsy and participants who are healthy, déjà vu might be thought of as a short-lived neurological event which leads to a disruption of recognition memory, whereby the sensations of memory retrieval become dissociated from memory retrieval itself: the decoupled familiarity hypothesis [1].

Less is known about recognition memory and déjà vu in other clinical populations, but there has been some exploration of the comparative prevalence of déjà vu in patients with epilepsy compared with affective disorders [3] and phobic anxiety–depersonalisation syndrome [4]. Both of these reports indicate that these groups report similar frequencies of déjà vu episodes when compared to patients with TLE. A recent review of TLE has called for further exploration of the link between clinical levels of anxiety and debilitating déjà vu, and suggested that further research is required to determine the nature of this relationship [1]. Here we discuss a case of a young man with anxiety and depersonalisation who reported experiencing persistent and debilitating déjà vu. There has been little consideration in the literature of psychogenic déjà vu: that is cases where the cause of the déjà vu seems to be psychological in nature (in this case probably the result of an anxiety disorder) [5].

## Case presentation

We present the case of a 23-year-old White British man who presented with persistent déjà vu in 2010. He reported experiencing these symptoms since early 2007 shortly after starting university. He had a history of feeling anxious, particularly in relation to contamination, which led him to wash his hands very frequently and to shower two to three times per day, and his anxiety worsened around the time he began university. Anxiety and low mood led him to take a break from university, and he then began experiencing déjà vu. His recollection of these early episodes was that they would last for minutes, but could also be extremely prolonged. For example, while on holiday in a destination that he had previously visited he reported feeling as though he had become ‘trapped in a time loop’. He reported finding these experiences very frightening.

He returned to university in 2007 and he described the déjà vu episodes as becoming more intense. He took lysergic acid diethylamide (LSD) once, and from then on the déjà vu was fairly continuous. In 2008 he was referred to specialists for neurological examination. Routine electroencephalogram (EEG) and magnetic resonance imaging were performed at a centre with experience in the diagnosis of epilepsy and were both normal. He was given a psychiatric diagnosis of depersonalisation and treated with a range of medications. His Dissociative Events Scale score (35.36) was abnormal (cut off =30) at the time of conducting the recognition memory task (October 2009).

He was assessed by AZ in 2010, at which point his persistent déjà vu caused him to avoid watching television and listening to the radio, as well as reading papers and magazines, as he felt he had already encountered the content before. His neurological examination was normal. At the time of assessment he reported a chronically low mood and felt anxious much of the time, although his compulsive behaviours were not a problem. There was a family history of obsessive compulsive disorder (OCD) on his paternal and possibly maternal side. Summary scores from his neuropsychological evaluation are presented in Table 1. His performance on the National Adult Reading Test was not dissimilar to that of controls, and his performance on the Wechsler Adult Intelligence Scale estimated his intelligence quotient as 112.

**Table 1:** **Patient and control group characteristics**

	Patient	Controls
	Score (z-score)	Mean (standard deviation)
Participant characteristics		
Age	23	21 (1.66)
Yrs Edu (mean)	15	15.25 (1.35)
NART (items correct)	35 (-1.29)	38.92 (3.04)
DES	35.36 (1.60)	20.11 (9.56)
CAPS	9	8.92 (5.36)
MOCI	9	–
DASS – Anxiety	–	7.82 (7.77) mild
DASS – STRESS	–	12 (8.15) normal
DASS – Depression	–	7.27 (8.40) normal
Recognition memory		
Discrimination index	21 (-0.72)	24 (4.15)
Remember hits	15 (-1.71)	21 (3.50)
Familiar hits	6 (0.39)	4.75 (3.20)
Guess hits	0 (-0.67)	1.17 (1.74)

*Abbreviations:* *CAPS* Cardiff Anomalous Perception Scale, *DASS-21* Depression Anxiety and Stress Scales, *DES* Dissociative Experiences Scale, *MOCI* Maudsley Obsessive-Compulsive Inventory, *NART* National Adult Reading Test, Years of Education (Yrs Edu).

In October 2009 we assessed his performance on a recognition memory task previously used with patients who report similar persistent déjà vu experiences/recollective confabulation (see [6], Experiment 3 for methodology). A comparison group of 11 male undergraduates conducted the same memory task (see Table 1 for sample characteristics). The control group were within the normal range for depression and stress as measured by the Depression Anxiety and Stress Scales, DASS-21, but were defined as having mild anxiety: note, lower cut-off is 7. They were also asked whether they had heard of déjà vu, frequency of déjà vu in the last month, and whether it impacted on their daily life. Eight controls had heard of déjà vu, one had not and two did not answer. Of the eight who had heard of déjà vu, only three had experienced it in the past month: two, three and 12 times. Of interest, the participant who reported approximately 12 déjà vu experiences within the past month scored highly on all subscales of the DASS, and his anxiety score was rated ‘extremely severe’, although he did not report that déjà vu impacted on his daily life. In short, the procedure for the memory task is as follows: participants study 30 words for an immediate test. They are then read a list of 60 words (30 studied words and 30 foils) and report whether each word is old or new. If classified as old, they report whether they remember it (can justify how they encountered it), find it familiar (they feel they have encountered it but cannot justify) or are guessing. We have previously demonstrated in dementia that people with persistent déjà vu-like experiences make very high levels of false positives (FPs), identifying new words as previously seen [6]. We have linked this deficit to a subtype of déjà vu, a disorder in the subjective experience of memory, as identified in the reports of ‘remembering’ and familiarity, which we termed déjà vecu. Déjà vecu is a particularly strong sensation of ‘re-living’ the present moment; it was this type of experience of which our case complained: rather than simply the unsettling feelings of familiarity which are normally associated with déjà vu, he complained that it felt like he was actually retrieving previous experiences from memory, not just finding them familiar.

Performance on the task is summarised in Table 1. Contrary to our expectations, our case made no FPs. Recognition memory was measured across all items and for all subjective states using a discrimination index, where FPs are subtracted from hits. His performance suggests he does not have a memory deficit, but his conservative performance (relatively low number of remember hits compared to controls and no guesses) suggests that he may be less confident in his memory ability than controls.

## Discussion

There is little scientific literature on any relationship between clinical levels of anxiety and déjà vu. Our case experienced high levels of anxiety and derealisation, and had a family history of OCD. There is no clear evidence in support of a neurological basis for his déjà vu, although we acknowledge that it is difficult to exclude this possibility absolutely and therefore do so with caution. A recent paper [7] reported the case of a 13-year-old girl who presented with persistent déjà vu but no clear symptoms of epilepsy. Detailed neurological examinations (EEG video monitoring) revealed the déjà vu feelings were auras associated with TLE seizures. This further supports the need for detailed investigations of patients presenting with what appears to be psychogenic déjà vu in order to rule out underlying neurological causes.

In contrast to patients with mild cognitive impairment (MCI) and dementia, our patient did not show an elevated level of FPs in his recognition memory. Critically, in comparison to these cases, he is fully aware of the false nature of his déjà vu experiences, and it is this awareness which presumably leads to the intact memory performance on the recognition memory test. Since he is metacognitively competent, he is not seduced by the feelings of false familiarity in his memory performance, even though he reported experiencing déjà vu during testing. Instead his performance appears to reflect a conservative strategy, perhaps arising from an appropriate mistrust of and reduced confidence in his memory abilities.

Whereas previous cases with déjà vu due to MCI and dementia have largely been anosognosic, our case is aware of the abnormal familiarity in his memory, and is in fact greatly distressed by it. This suggests two dimensions along which déjà vu experiences can vary: awareness and distress. In this psychogenic case, our patient is similarly aware of the unreality of his experiences and they are constantly accompanied or caused by pathological levels of anxiety. In TLE, déjà vu is also interpreted as a memory error, and the epilepsy does not produce anosognosia. Déjà vu experiences are frequently distressing to patients with TLE, but in established cases the explanation for the experience is known (abnormal firing in the temporal lobe). In relation to our case, distress caused by the déjà vu experience may itself lead to increased levels of déjà vu: similar feedback loops in positive symptoms are reported in other anxiety states (e.g. panic attacks [8]).

It is plausible on neurobiological grounds that anxiety might lead to the generation of déjà vu. The hippocampal formation, a structure of central importance in declarative memory and the ability to engage in recollection, is also implicated in anxiety as part of the septo-hippocampal system [9]. Although this report does not prove a link between anxiety and déjà vu, it does further support the suggestion that this area is worthy of further investigation.

## Conclusions

In sum, we have reported the case of a 23-year-old man who appears to display a form of psychogenic déjà vu, without a gross impairment of memory, without epileptic features and without producing high levels of FPs on a memory task that has revealed abnormal processes in patients with persistent déjà vu and neurodegenerative disorder. He reports high levels of anxiety, probably both contributing to and resulting from his frequent experience of déjà vu. This case study contributes to the limited literature on the relationship between anxiety and déjà vu, and highlights the need for further investigation of the occurrence of déjà vu in psychiatric disorders.

## Consent

Written informed consent was obtained from the patient for publication of this case report and any accompanying images. A copy of the written consent is available for review by the Editor-in-Chief of this journal.

## Abbreviations

DASS-21: Depression Anxiety and Stress Scales
EEG: Electroencephalogram
FPs: False positives
LSD: Lysergic acid diethylamide
MCI: Mild cognitive impairment
OCD: Obsessive compulsive disorder
TLE: Temporal lobe epilepsy.
